# Xylazine (veterinary sedative) use in Puerto Rico

**DOI:** 10.1186/1747-597X-6-7

**Published:** 2011-04-11

**Authors:** Rafael A Torruella

**Affiliations:** 1 National Development and Research Institutes, Inc. (NDRI) and Public Health Solution (PHS) 71 West 23rd Street, New York, NY 10010, USA

## Abstract

Human xylazine use in Puerto Rico merits particular attention for its unprecedented scale and depth. Although Puerto Rican injecting drug users (IDUs) have reported using this drug since the early 2000s, little is being done in the research and service delivery sectors as it is claimed that xylazine severely impacts the health of its users. This report provides information on xylazine use and its associated harms. Data from one semi-structured interview collected in New York City (2007-2008) as part of a larger research study with migrant Puerto Rican drug users is presented as a case study. Xylazine, a veterinary sedative, is an adulterant and complement to other drugs and its chronic use is reported to be associated with physical deterioration. Because human xylazine use has been reported in other locations outside of Puerto Rico, this substance could also emerge as an adulterant in other markets to the levels currently experienced in Puerto Rico. Research and interventions are needed to provide adequate services on the island, better understand how the use of xylazine affects its users, and to reduce the possibility of increased xylazine use on the state-side.

## Findings

Since the early 2000's Puerto Rico has seen an emergence of a substance among its injecting drug users (IDUs) called xylazine. Xylazine is a non-opiate sedative, analgesic, and muscle relaxant certified exclusively for veterinary use. The harms associated with the chronic use of xylazine, or "*Anestesia de Caballo*" (Horse Anesthetic) as it is commonly known on the streets of Puerto Rico, are not well documented as it has received little attention from researchers. Yet, some associations have been made between the use of xylazine and open skin ulcers [1] that have serious negative effects on the daily lives of injecting drug users. While the precise reasons why xylazine emerged in the island's drug market are still unknown, it is argued that the same motivating factors could also occur in other markets, including those in the continental United States and create an equally problematic public health issue.

Having worked in the field of drug use for many years as a direct service provider and as a researcher, this author directly witnessed many of the harms associated with xylazine use. This short report is framed by those service provision experiences on both the island and the state-side as well as by this researcher's studies in injection drug use. The goal of this report is to present information on human xylazine use and its associated harms. In turn, this information highlights the need to generate additional research and adequate services to reduce these drug-related harms. Illustrations of how xylazine is viewed from a drug user's perspective are presented in data from an interview conducted for a larger study. The specific interview was chosen out of a small number that discussed xylazine use in Puerto Rico because of the participant's extensive experience with the substance and his detailed and comprehensive description of these experiences. Xylazine use was not a main topic in the interview protocol, therefore the topic was only discussed by some research participants. The lager study was the author's dissertation research (UMI No. 3396474) and data was collected between 2007 and 2008 with Puerto Rican injecting drug users who relocated from Puerto Rico to New York City for drug treatment services. Human subjects approval was attained from the City University of New York's Graduate Center IRB. A first wave of anonymous ethnographic data was collected through participant observation. Informed by the contacts and knowledge afforded by the participant observation, a second wave of data was collected utilizing semi-structured interviews (N = 13) for which all participants signed consent forms and were audio recorded. Confidentiality was further assured to participants by securing an NIH-issued Certificate of Confidentiality.

While studies on the (mis)use of xylazine on humans exists [2-6], there is a dearth of research on how this substance becomes diverted to the illicit drug market, the context of use, and the harms related to its chronic use. One study [7] detected xylazine and fentanyl (the former being an veterinary sedative/anesthetic and the latter a synthetic opiate used for the medical treatment of pain in humans) in drug related deaths in Philadelphia, Pennsylvania and suggested that both drugs could be used as adulterants in heroin. While the precise reasons why these drugs are used as adulterants are unknown, the authors mentioned that "financial concerns, e.g., increased street sale value, and enhanced drug effects may be some of the driving forces" (p.497). More importantly, they advocate for research on xylazine and its effect on humans because: "it is a veterinary drug and is not approved for human use. [...] Unfortunately, its use in humans can be fatal, especially in overdose cases." (p.498)

Xylazine was confirmed as the anesthetic substance used in Puerto Rico by testing exchanged needles in 29 sites in 11 municipalities [1]. The authors of the study, beyond asserting that the substance was not "confined to a small area in Puerto Rico" (p. 292) mentioned that xylazine was highly prevalent in syringes collected in cattle-farming towns (e.g., Arecibo, Yauco, and Guanica: 98%, 100%, and 100% respectively). Noteworthy is that "IDUs whose syringes contained Xyz were also more likely to complain of poor health than those whose syringes did not, [...] [t]he former also had higher incidence of skin ulcers than the latter" (p.292). Interestingly, their data did not point to homelessness as having a significant impact on skin ulcers. Research such as this is critical as it begins to tease apart the negative effects of the substance itself from those effects of the contextual factors that condition the use of xylazine. The study also stressed some points where xylazine-related information is needed: 1) its distribution, 2) its chronic use and its association to physical deterioration, and 3) how widespread xylazine abuse is beyond the island.

Similarly, and unpublished study by Reyes JC, Robles R, Negrón J, Colón H, Matos TD, and Calderón JM titled *The Emerging of Xylazine as a New Drug of Abuse and its Health Consequences Among Drug Users in Puerto Rico *found a prevalence of xylazine use in prior 30-days of 80.7% among its sample of 89 drug users recruited from 12 communities from the San Juan metropolitan area. Xylazine users were more likely to be male, under the age of 30, living in a rural area, and injecting versus inhaling xylazine. Additionally, 35.2% reported skin lesions, 28.2% reported an increase in injection use, and 21.1% reported at least one overdose episode.

### Xylazine as an adulterant and complement to other drugs: Distribution

Pipo (pseudonym), a savvy consumer, describes how he first encountered xylazine and how it was marketed in Puerto Rico; first as an adulterant to heroin packaged for the consumer to control the ratio him or herself and later in pre-mixed form.

I opened up the bag and when I threw it in the cooker, a really fucking strong medicine smell. [...] That was the first heroin that they cut with *anestesia *(xylazine) in Puerto Rico, the "Blue Demon". [...] What those people did was that they would place heroin inside, they would then fold the paper the first time, the first fold, there outside they would put the *anestesia *and then the fold. They would call that, *el regalito *(the small gift). And when you would open the bag, you would open the first fold and there was a half a bag [...] [of] *anestesia *and you would put some. And when you would open [the rest of] the bag you would put the other [heroin]. [...] It was later when they started to mix it, [...] 80% *anestesia *and 20% heroin.

Pipo indicated that xylazine is marketed in the same locations as heroin and it has been sold in packages together, but not mixed, with heroin as well as in pre-mixed form. Non-mixed packages allow users to control the heroin/xylazine ratio - and therefore controlling some of the substances' effects on the user.

Another non-mixed packaging form, presumably adjusting to users' demand who often seek a balance between the "down" of both the heroin and xylazine, added cocaine. This packaging form was called "*el combito*" (the small combo):

"They put coke, a $3 of coke. They put a $3 of heroin. And an *anestesia *[xylazine] of $2. You know, heroin [and] the *anestesia *are pure."

In essence, *el combito *is a speedball - a mix or heroin and cocaine - with xylazine package for $8 with each substance kept separate for the user to tailor to her/his liking.

Where xylazine and heroin are sold pre-mixed, adulterating the heroin with xylazine becomes a sought after skill as it involves taking xylazine in its original liquid form and mixing it with heroin without damaging either substance. This was reported to be done locally so it could be packaged and sold as soon as the mixture was ready.

### Chronic use and physical deterioration

Two key issues about the chronic use of xylazine are the physical dependence to the substance and the noticeable open skin ulcers it is claimed to create. Pipo here begins by describing the first time he used xylazine and his later physical dependence on both heroin and xylazine along with his need to avoid withdrawal symptoms:

I shot the *anestesia *[...] and I felt asleep face first and when I opened my eyes five hours had gone by and I was laying on the floor. [...] I don't remember anything. I don't remember anything! I fell down and I was gone. And I said: What the hell is this?! Oh, and I woke up sick [withdrawing]!

[...]

I get there and don't cop just heroin. I cop *anestesia*. Because that it what is going to get me high and what is going to get me straight [and reverse withdrawal symptoms]. I am not going to waste my money in just heroin because I'm going to stay the same. Do you understand? I'm going to stay the same.

Pre-mixed with heroin or not, xylazine users claim that chronic use of this substance creates open skin ulcers. Yet, the author only found one published study [1] and one unpublished poster presentation that explored this important association between xylazine and skin ulcers. Among the drug using community, these open ulcers are referred to as abscesses.

[T]he times when the abscesses [...] started to appear, I would come here, to the United States. [...] [When the abscesses began to appear] I already knew. [...] I had seen them [before]. [...] [T]here are people that take a longer time in blowing up [with abscesses] than others. [...] I am one in which it took a while. But when I saw that people were rotting I would get scared because I always have said that I am a junky with style.

These ulcers are a serious health concern for drug users for several key reasons. First, they are painful. This promotes further injections in the injection/ulcer site with xylazine (functioning as a sedative/anesthetic). This subsequently creates a cycle that heightens the need for medical attention and adequate treatment. It should be noted, however, that not all ulcers appear in the injection sites, they are also reported to appear in non-injection site extremities. Second, these open skin ulcers emit a strong odor, ooze, and in severe cases limit the mobility of the extremities. In some cases, amputations have been performed on the affected extremity/extremities. Third, xylazine users attest that when they have asked for help from clinics on the island, health professionals commonly deny services because of their ulcers. Pipo, in the above interview mentions that he was lucky because the ulcers took time to develop in his body - by the time they developed, he had relocated to the state-side and could access medical services. He points to the lack of treatment for these ulcers in Puerto Rico, the lack of xylazine availability on the state-side, and the knowledge that some state-side hospitals have gained by providing services to drug users that are relocated from Puerto Rico to the state-side for treatment by the local Puerto Rican government.

With no specialized treatment services for xylazine users on the island, except for some community based organizations who attend to xylazine user's basic healthcare needs (e.g., medical/wound care, syringe exchange, education), these open ulcers grow to emit a strong odor and look severe to both the medically trained and the un-trained eye. The combination of the "basic" stigma towards drug users [8] compounded with the olfactory and visual severity of these open skin ulcers (re)stigmatize and further socially exclude these drug users. A colleague said it best when she saw the ulcers and the effects these had on non-users in the island: "injecting drug users are being treated as if they were lepers."

### The spread of xylazine

Because human xylazine use has been reported in other locations and not just in Puerto Rico (e.g., Philadelphia) it stands that this substance could also emerge as an adulterant in other markets to the levels currently experienced in Puerto Rico. Surely, the scarcity of treatment services for drug users on the island in comparison to the state-side [9] and the current trend of local elected official in Puerto Rico relocating IDU to the state-side for drug treatment services does not aid to contain this situation to the island. Yet, ignoring the emergence of a substance like xylazine will not result in sound public health responses and will only add to the detriment of drug users and society as a whole. Specialized services and research need to be dedicated to xylazine users - and Puerto Rican IDUs in general. Meanwhile, tracking the use of xylazine on the state-side markets (without negatively drug-profiling Puerto Rican communities) seems like a pertinent first step, as xylazine is an already known adulterant, albeit only in Puerto Rico has it been used to these alarming levels.

## Competing interests

The author declares that they have no competing interests.

## Author's information

RT's work bridges the interrelated - yet often discrepant - areas of drug use research and drug use service delivery. RT recently earned a Ph.D. in social-personality psychology from The Graduate Center, City University of New York (CUNY) while actively working with the service delivery sector in New York City. RT is currently a National Institute of Drug Abuse (NIDA) funded post doctoral fellow at the National Development and Research Institutes, Inc. (NDRI) and contributes as board member at New York Harm Reduction Educators, Inc. (NYHRE).
